# QUANTITATION OF MAST CELLS AND COLLAGEN FIBERS IN SKIN TAGS

**DOI:** 10.4103/0019-5154.57605

**Published:** 2009

**Authors:** Omar Soliman El Safoury, Marwa M Fawzy, Zeinab M El Maadawa, Dalia H Mohamed

**Affiliations:** *From the Department of Dermatology, Cairo University, Egypt*; 1 *From the Department of Histology, Cairo University, Egypt*

**Keywords:** *Image analyzer*, *mast cells*, *mean collagen area %*, *skin tags*, *Bismarck brown*

## Abstract

**Background:**

Skin tags are common benign skin tumors usually occurring on the neck and major flexors of elder people.

**Aims:**

The aim of this study is to perform quantitation of mast cells and collagen fibers in skin tags and normal skin in diabetics and nondiabetics, to find a possible correlation between mast cells and collagen fibers in the pathogenesis of skin tags.

**Methods:**

Thirty participants with skin tags were divided into two groups (15 diabetic and 15 nondiabetic). Three biopsies were obtained from one anatomical site: A large skin tag, a small skin tag, and adjacent normal skin. Mast cells stained with Bismarck brown were counted manually in ten different fields of each section with magnification ×1000 and the average count was correlated with the percentage of mean collagen area in five fields done by the image analyzer.

**Results:**

A statistically significant correlation between mast cell count and percentage of collagen mean area was detected in both studied groups (except in large skin tags of the nondiabetic group).

**Conclusion:**

The positive correlation between mast cell count and percentage of collagen mean area suggests the critical role of mast cells in the etiogenesis of skin tags through its interaction with fibroblasts.

## Introduction

Skin tags are small flesh colored to dark brown sessile or pedunculated papillomas commonly occuring on the neck, frequently seen in axilla and eyelids, less often on the trunk and groin. Both sexes have the same incidence.[1] Histologically, they are composed of loose collagen fibers and dilated capillaries.[2] Skin tags has been reported to be associated with many diseases, the list includes diabetes mellitus,[3] obesity,[4] acromegaly,[5] Crohn's disease,[6] aging,[7] child abuse,[8] organ transplants,[9] and colonic polyps.[10] It was also recorded with pregnancy[11] and human papilloma virus.[12] In 2007, Zaher *et al*.[13] recorded an increase in mast cell count in skin tags than normal skin.

The aim of this study is to perform quantitation of mast cells and collagen fibers in skin tags and normal skin in diabetics and nondiabetics, to find a possible correlation between mast cells and collagen fibers in the pathogenesis of skin tags.

## Materials and Methods

For this study, 30 participants attending the outpatient clinic seeking for the removal of their skin tags were divided into two groups:

Group I: This group consisted of 15 nondiabetic participants, four males and 11 females with ages ranging between 25 and 55 years with a mean of 41.67 ± 9.9 years.

Group II: This group consisted of 15 diabetic participants, six males and nine females with ages ranging between 37 and 75 years with a mean of 53±10.29 years. Following general skin examination, the number, site, color, shape, and consistency of skin tags were recorded. The random blood sugar estimation for all participants was performed and documented. From each participant, three biopsies were taken in the same area (neck) as follows: Large skin tag (length > 4 mm, small skin tag (length < 2 mm), and normal skin (as a control). For local anesthesia, 1% xylocaine was injected around the area to be biopsied. Skin biopsy specimens were taken with a punch biopsy instrument 4 mm in diameter. Biopsies were fixed in 10% formalin solution for 24 h. All biopsies were processed by routine paraffin technique. Sections of 4 μm in thickness were cut as near the middle of the biopsy as possible and perpendicular to the skin surface. Each section was stained with Bismarck brown stain for mast cells[14] and Masson's trichrome stain for collagen fibers.[15]

### Mast cell count

Mast cells were counted manually in ten different fields of each section using an Olympus microscope with magnification ×1000. All mast cell profiles were counted. All counts were done by the same doctor. Masson's trichrome stained collagen fibers in the form of dense green deposits of a fixed degree were submitted for image analysis using Leica Imaging System, Ltd., Cambridge, England (Leica Qwin), which is formed of a personal computer connected to a microscope. The image analyzer was first calibrated to convert the measurement unit produced by the image analyzer program (pixels) into actual micrometer units. Five fields from each section were selected and the area percent of the dense green deposits were measured by the image analyzer in relation to a standard measuring frame which was 7286.8 μm^2^ using magnification ×400. The area percent obtained from the image analyzer was subjected to statistical analysis.

### Statistical analysis

Data were statistically described in terms of range, mean ± standard deviation (±SD), median, frequencies (number of cases), and relative frequencies (percentages) when appropriate. The comparison of quantitative variables between the study groups was done using Mann–Whitney U test for independent samples. The comparison of different groups within each group was done using Freidman analysis of variance (ANOVA) test with posthoc multiple two-group comparisons. For comparing categorical data, Chi square (χ^2^) test was performed. Exact test was used instead when the expected frequency is less than 5. A probability value (*P* value) less than 0.05 was considered to be statistically significant. All statistical calculations were done using computer programs Microsoft Excel version 7(Microsoft corporation, NY, USA). SPSS (Statistical Package for the Social Science: SPSS Inc., Chicago, IL, USA) and Arcus QuickStat (Biomedical), (Arcus Statistical Software, Research Solutions, Addison Wesley Longman Ltd. USA).

## Results

### Comparison between mast cell count in skin tags and normal skin in both studied groups

**Figure 1:**
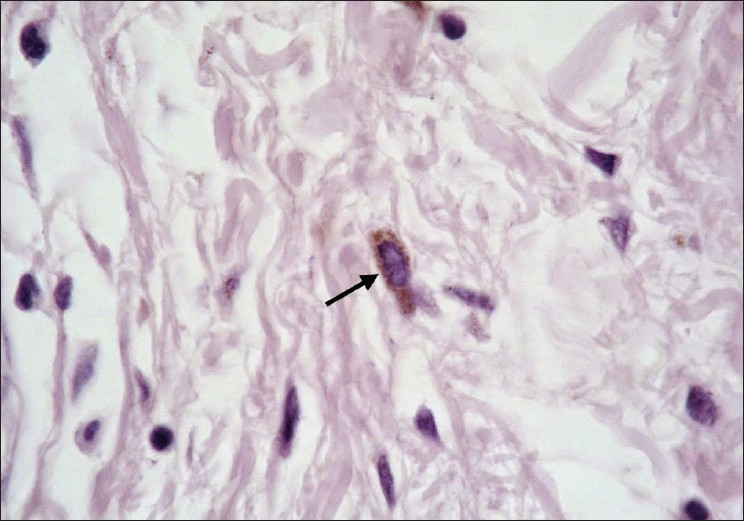
Skin tag in a non diabetic participant showing a brown stained mast cell (arrow) (Bismarck brown, ×1000)

**Figure 2:**
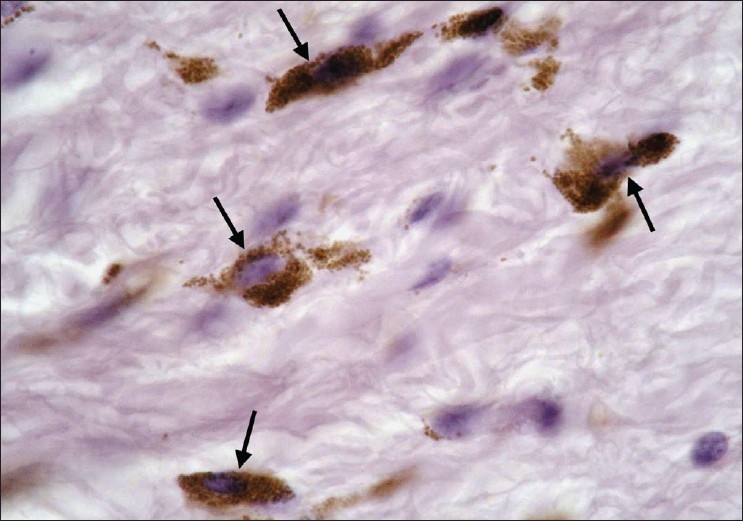
Skin tag in a diabetic participant showing a brown stained mast cell (arrow) (Bismarck brown, ×1000)

Mast cell count in large ST of diabetic group was statistically higher than the nondiabetic group *P* value = 0.001 (*P* < 0.05). Similarly, mast cell count in small ST of diabetic group was statistically higher than the nondiabetic group *P* value = 0.035 (*P* < 0.05). Mast cell count in control normal skin of diabetic group was statistically higher than the nondiabetic group *P* value = 0.001 (*P* < 0.05) [Table 1] [Figures 1 and 2] .

**Table 1:** Comparison between mast cell count in skin tags and normal skin in both studied groups

Study population		Large ST	Small ST	Normal skin
	No. of cases	15	14	15
Group I	Mean	7.33	9.86	7.47
(Non DM)	SD	3.68	5.59	2.9
	Maximum	18	22	14
	Median	7	8	6
	Minimum	3	4	4
	No. of cases	14	14	15
Group II	Mean	17.64	15.64	13.73
(DM)	SD	6.89	7.09	3.13
	Maximum	26	25	21
	Median	19	16.5	14
	Minimum	3	1	9
Analysis	*P* value	0.001	0.035	0.001

### Comparison between the percentage of collagen mean area in skin tag lesions and control normal skin in both studied groups

**Figure 3:**
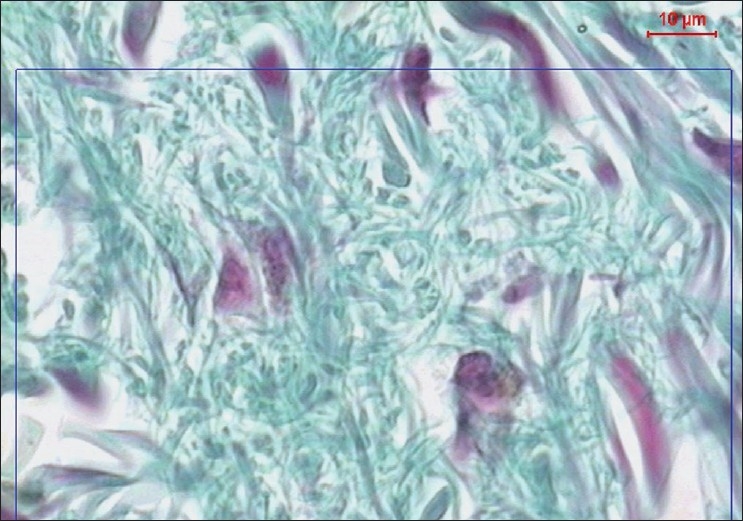
A copy of display on the monitor's screen of the image analyzer in non diabetic participant showing collagen fibres in green color (Masson's trichrom, ×400)

**Figure 4:**
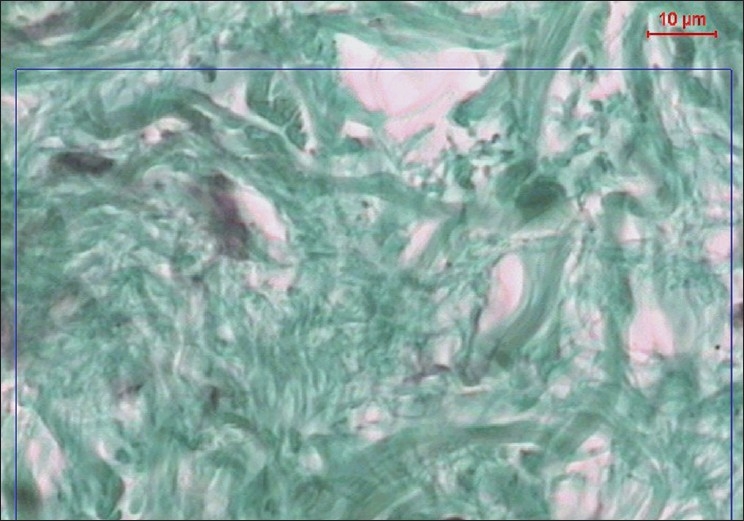
A copy of display on the monitor's screen of the image analyzer in diabetic participant showing collagen fibres in green color (Masson's trichrom, ×400)

The percentage of collagen mean area in large ST of diabetic group was statistically higher than the nondiabetic group *P* value = 0.002 (*P* < 0.05). Although the percentage of collagen mean area in small ST of diabetic group was higher than the nondiabetic group yet, it was non significant *P* value = 0.214 (*P* > 0.05).The percentage of collagen mean area in control normal skin of diabetic group was statistically higher than the nondiabetic group *P* value = 0.001 (*P* < 0.05) [Table 2] [Figures 3 and 4].

**Table 2:** Comparison between percentage of collagen mean area in skin tag lesions and control normal skin in both studied groups

Study population		Large ST	Small ST	Normal skin
	No. of cases	14	14	12
Group I	Mean	20.33	25.16	16.59
(Non DM)	SD	10.63	13.95	7.29
	Maximum	48.56	46.97	37.12
	Median	16.05	16.38	14.54
	Minimum	10.23	10.23	10.23
	No. of cases	14	14	15
Group II	Mean	38.97	36.6	31.2
	SD	14.66	18.29	8.66
	Maximum	68.62	73.86	45.56
	Median	38.72	35.39	31.63
	Minimum	13.47	12.6	16.05
Analysis	*P* value	0.002	0.214	0.001

### Correlation between mast cell count and percentage of collagen mean area in both groups

#### In the nondiabetic group

There was statistically significant correlation between mast cell count and percentage of collagen mean area in small tags and control normal skin *P* values = 0.001 and 0.012, respectively (*P* < 0.05), while in large tags, there was no statistically significant correlation between mast cell count and the percentage of collagen mean area *P* value = 0.052 (*P* > 0.05) [Table 3].

**Table 3:** Correlation between mast cell count and percentage of collagen mean area in the nondiabetic group

		Mast cell-large tag	Mast cell-small tag	Mast cell-control
Collagen mean area%-large tag	Correlation coefficient	0.550	0.096	0.306
	Sig (2- tailed)	0.052	0.767	0.309
	N	13	12	13
Collagen mean area%-small tag	Correlation coefficient	−0.045	0.926	0.091
	Sig (2- tailed)	0.880	0.001	0.758
	N	15	13	14
Collagen mean area%-Control	Correlation coefficient	0.332	−0.023	0.694
	Sig (2- tailed)	0.292	0.946	0.012
	N	12	11	12

Sig (2– tailed) = *P* value

#### In the diabetic group

There was statistically significant correlation between mast cell count and the percentage of collagen mean area in large tags, small tags, and control normal skin *P* values = 0.001, 0.001, and 0.001, respectively (*P* < 0.05) [Table 4].

**Table 4:** Correlation between mast cell count and percentage of collagen mean area in the diabetic group

		Mast cell-large tag	Mast cell-small tag	Mast cell-control
Collagen mean area%-large tag	Correlation coefficient	0.830	0.140	0.056
	Sig (2- tailed)	0.001	0.647	0.850
	N	14	13	14
Collagen mean area%-small tag	Correlation coefficient	−0.040	0.893	0.190
	Sig (2- tailed)	0.897	0.001	0.515
	N	13	13	14
Collagen mean area%-Control	Correlation coefficient	−0.040	0.194	0.796
	Sig (2- tailed)	0.893	0.505	0.001
	N	14	14	15

Sig (2– tailed) = *P* value

## Discussion

Zaher *et al*.,[13] reported a significant increase in mast cell count in skin tags in comparison to normal skin in both diabetic and nondiabetic participants. In this study, mast cell count was higher in diabetic than nondiabetic participants. It should be noted that mast cell count differs by using different stains. In 1987, Marshall *et al*.,[16] noticed that more mast cells were visualized with alcian blue / safranin than toluidine blue in human dermis. Also in 2000, Florenzano and Bentivoglio[17] reported that Pinacyanol, alone or with erythrosine, stained is more sensitive than toluidine blue (in rat thalamus).

Generally, mast cells participate in the pathogenesis of fibrotic diseases, they are found to stimulate fibroblast proliferation and collagen synthesis through some fibrotic mediators such as histamine and tryptase.[18] Mast cells are intimately associated with fibroblasts in tissues. Reciprocally, fibroblasts maintain mast cells *in vivo*. After co-culture with fibroblasts, immature mast cells changed to mature mast cells capable of synthesizing heparin and proteoglycans as well as changing their phenotype to resemble connective tissue type mast cells.[19] A study of the effect of human mast cells on fibroblast activity using an organotypic skin- equivalent culture system (mast cells in a collagen gel with neonatal dermal fibroblasts) revealed that there was increased synthesis of type a1(I) pro-collagen mRNA in presence of mast cells especially if degranulated. Mast cell tryptase stimulated fibroblast chemotaxis and also stimulated collagen messenger ribonucleic acid mRNA synthesis.[20] Clinically, Jones *et al*.,[21] found that there were an increased number of mast cells in diabetic nephropathy associated with renal fibrosis.

The correlation between mast cell count and percentage of collagen mean area in our study was statistically significant in both studied groups, except in large lesions of the nondiabetic group, but it was nearly significant. To our knowledge, no other studies demonstrated the correlation between mast cell count and percentage of collagen mean area in skin tags. However in 2006, Facoetti *et al.,*[22] used similar methodology in cardiologic research, their work was on rat cardiac muscle mast cells. Mast cell count was done and percent area of interstitial collagen deposits in 10 random fields was determined by the Image analyzer. Their data demonstrated a close relationship between rat cardiac muscle mast cell activation and collagen deposition.

Finally, we conclude that mast cells may play a critical role in the pathogenesis of skin tags through interaction with fibroblasts by increasing the collagen area.
